# Tag-free, specific conjugation of glycosylated IgG1 antibodies using microbial transglutaminase†

**DOI:** 10.1039/d2ra05630e

**Published:** 2022-11-22

**Authors:** Adem Hadjabdelhafid-Parisien, Sebastian Bitsch, Arturo Macarrón Palacios, Lukas Deweid, Harald Kolmar, Joelle N. Pelletier

**Affiliations:** Department of Biochemistry, University of Montreal Montreal QC Canada; Center for Green Chemistry and Catalysis (CGCC) Montreal QC Canada; PROTEO, the Quebec Research Network on Protein Function, Engineering and Applications Quebec City QC Canada; Institute for Organic Chemistry and Biochemistry, Technical University of Darmstadt Darmstadt Germany Harald.Kolmar@TU-Darmstadt.de; Centre for Synthetic Biology, Technical University of Darmstadt Darmstadt Germany; Department of Chemistry, University of Montreal Montreal QC Canada joelle.pelletier@umontreal.ca

## Abstract

We present an efficient approach for tag-free, site-specific conjugation of a fully glycosylated antibody using microbial transglutaminase (mTG). We created variants of trastuzumab where a single surface-exposed residue of the human crystallizable fragment had been substituted to glutamine, with the objective of enabling site-specific mTG-mediated conjugation with primary amine payloads. MTG reactivity was determined by conjugation to an amino fluorophore, demonstrating effective tag-free conjugation at the newly introduced I253Q site. The conjugation of one payload per antibody heavy chain was confirmed by mass spectrometry. We further demonstrated two-step mTG/click chemistry-based conjugation of I253Q trastuzumab with monomethyl auristatin E. Cytotoxicity and specificity of the resulting antibody–drug conjugate were indistinguishable from trastuzumab conjugated by another method although binding to the neonatal Fc receptor was impaired. The resulting fully glycosylated ADC is unique in that it results from minimal modification of the antibody sequence and offers potential for application to cellular imaging, fluorescence microscopy, western blotting or ELISA.

## Abstract 2

Substitution I253Q on a glycosylated IgG1 antibody allows microbial transglutaminase-mediated conjugation of a fluorophore or a clickable auristatin. The resulting antibody–drug conjugate showed excellent cell toxicity and no FcRn-mediated recycling.

## Graphical abstract

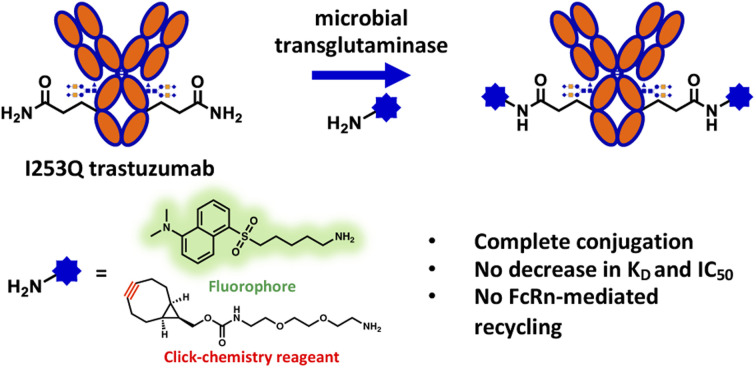

## Introduction

Site-specific protein modification is increasingly applied to synthesize protein conjugates for a wide array of applications.^1,2^ Applications of conjugated proteins have been extended by site-selective modification, for example using a dual-labeled protein for FRET imaging or to improve biodistribution and pharmacokinetics in the context of therapeutic proteins.^3,4^ Site-specific modification contrasts with non-specific conjugation methods such as conjugation to canonical lysine or cysteine residues. Non-specific modification produces heterogeneous composition with respect to degree of conjugation (DoC) of the target protein as a result of insufficient difference in reactivity of these residues. Conjugation at undesirable sites can lead to loss of protein function, and batch-to-batch heterogeneity is a concern. Those factors are of particular importance in the case of antibody conjugation; in addition, a high DoC increases aggregation of antibody conjugates.^5–7^

Various strategies have been developed to enzymatically conjugate antibodies and thus increase batch-to-batch homogeneity by targeting specific residues.^8,9^ One such enzymatic strategy entails the use of microbial transglutaminase (mTG) from *Streptomyces mobaraensis*. MTG natively produces an isopeptide bond between protein-borne glutamine and lysine residues. *In lieu* of a lysine residue, a wide variety of molecules can be used as amino substrates for protein conjugation. Polymers, small organic compounds and proteins have been conjugated using mTG for different applications.^10–13^ The isopeptide bond formed by this enzyme is stable in circulation and resistant to proteolysis, which represents an advantage for therapeutic applications.

**Fig. 1:**
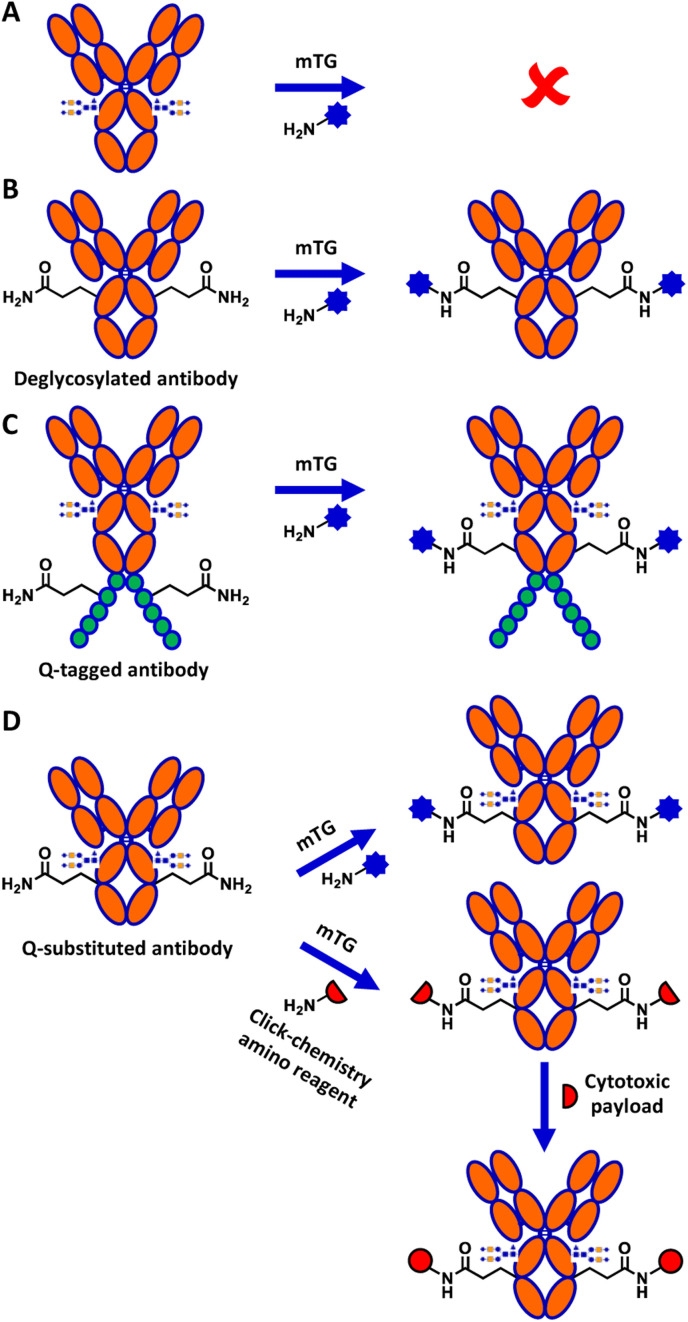
Overview of mTG-mediated glutamine conjugation of antibodies. (A) Glycosylation masks the unique, natively reactive glutamine of an IgG1 antibody, Q295. (B) Deglycosylation exposes Q295 which can then be used as a glutamine substrate for mTG conjugation, producing a deglycosylated conjugate. (C) Glutamine-tags (Q-tags, typically ≥4 residues) have been developed for conjugation of glycosylated antibodies. (D) This work: glutamine substitutions are screened for reactivity with mTG. Top reaction: direct conjugation with an aminated substrate; bottom reaction: mTG-mediated conjugation of an aminated reagent is followed by click chemistry to install a cytotoxic payload.

Although mTG is widely used in biotechnology, the mechanism explaining its reactivity toward specific glutamine residues relative to others is not well understood.^14^ Of particular interest, despite IgG1 human crystallizable fragment (hFc) antibody displaying 8 surface-exposed glutamines,^15^ none shows reactivity towards mTG in the context of the native, glycosylated antibody (Fig. 1A). Conjugation to the surface-exposed Q295 glutamine residue that is conserved in the Fc of IgGs was previously reported for IgG1 (Fig. 1B).^16^ However, that process required deglycosylation of the neighbouring N297 residue. This requirement is detrimental for many applications of antibody conjugates since glycosylation is an important contributor to antibody stability and solubility.^17^

Several strategies have been developed to circumvent the need to deglycosylate the antibody prior to reaction with mTG. These include grafting a short glutamine-containing peptide (Q-tag) recognized by mTG as a substrate (Fig. 1C), the directed evolution of mTG to modify its reactivity, or direct conjugation on lysine residues of the antibody.^18–20^ However, the incorporation of Q-tags can disrupt the properties of the antibody and result in undesired immunogenic potential in the context of therapeutic applications, whereas use of the antibody as a direct amino substrate of mTG limits conjugation partners to reactive glutamine-containing peptide derivatives.^21^ Antibody conjugation to amino partners with mTG would circumvent these limitations.

We previously reported the glutamine-walk method to screen for reactive glutamine-containing substrates for mTG conjugation, following the substitution of single amino acids into glutamine in different proteins.^11,22^ This method was applied to the hFc of IgG1 antibodies. The hFc had been expressed in *E. coli* SHuffle T7 Express to allow for assembly of dimeric hFc by disulfide bridging. Two highly reactive IgG1 hFc variants, I253Q and Y296Q, were identified as effective mTG substrates for conjugation with amino fluorophores, suggesting that they may enable tag-free conjugation in the context of a complete, glycosylated antibody.^22^

Here, we demonstrate successful direct conjugation to glycosylated trastuzumab antibody produced in mammalian cells with an amino fluorophore, confirming that specific reactivity of mTG to the newly engineered site was maintained in the context of full-length antibody. We further demonstrate mTG-mediated chemoenzymatic conjugation of a cytotoxic payload onto trastuzumab (Fig. 1D). The resulting antibody–drug conjugate (ADC) was validated for its binding affinity and its cytotoxicity, demonstrating the applicability of this approach to conjugate glycosylated antibodies with minimal sequence alteration.

## Results and discussion

Using the glutamine-walk approach, we previously identified two highly reactive mTG substrates, the I253Q and Y296Q variants of IgG1 hFc. Conjugation of these hFc variants with the amino fluorophore dansylcadaverine by mTG resulted in a 3 to 4-fold increase in conjugation efficiency as evaluated by fluorescence intensity following resolution by SDS-PAGE.^22^ Here, we verified whether the newly introduced glutamines maintain their reactivity for conjugation with mTG in the context of a full-length glycosylated antibody. The human epidermal growth factor receptor-2 (HER2)-targeting antibody, trastuzumab, was chosen because it is a well-characterized therapeutic IgG1 antibody. Seven glutamine variants were selected according to the reactivity they conferred in the context of the IgG1 hFc. In addition to the two highly reactive I253Q and Y296Q hFc variants (respectively exhibiting a 3 and 4-fold increase with respect to the control reaction containing no mTG), we also selected the non-reactive hFc variant, S254Q (0.9-fold increase), as a negative control and four weakly reactive hFc variants (M252Q, V282Q, K340Q and P445Q; fold-increase between 1.1–1.5) to further verify whether reactivity in the IgG1 hFc will be reflected in the context of the entire IgG1 antibody. The selected trastuzumab glutamine variants were expressed and purified. Purification profiles were all similar to the wild type (WT) trastuzumab in yield and elution profile, suggesting similar soluble folding (Fig. S1†). Purification typically yielded 0.8–1.7 mg from a 30 mL culture (Table S2†).

**Fig. 2:**
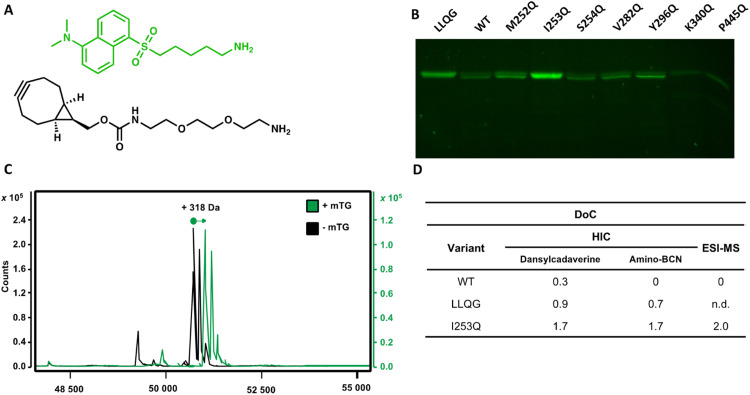
Screening glycosylated trastuzumab with newly introduced glutamines for mTG conjugation with amino substrates. (A) The amino substrate dansylcadaverine was monitored according to its fluorescence. Amino-BCN served as a strained alkyne in SPAAC. (B) Resolution of trastuzumab variants with newly introduced glutamine for mTG-mediated conjugation of dansylcadaverine. The WT glycosylated trastuzumab served as a negative control for reactivity. (C) Mass spectra of reduced I253Q trastuzumab heavy chain conjugated to dansylcadaverine (+mTG, green line) compared to reaction in absence of mTG enzyme (−mTG, black line). A mass increase of 318.5 Da is expected upon conjugation to dansylcadaverine. (D) DoC determined by HIC or by ESI-MS for the conjugation of WT, LLQG-tagged or I253Q trastuzumab with either dansylcadaverine (HIC and ESI-MS) or amino-BCN (HIC only). A DoC of 2 is expected upon conjugation of one molecule of amine on each heavy chain of trastuzumab.

Conjugation with dansylcadaverine by mTG was performed as previously reported and analyzed by SDS-PAGE to rapidly visualize the reactivity of each trastuzumab variant.^22^ The I253Q trastuzumab variant displayed the highest labeling efficiency (Fig. 2) (4.9-fold increase relative to WT signal). The Y296Q substitution led to poor mTG labeling of the glycosylated antibody with dansylcadaverine (1.8-fold WT signal). The weak reactivity at this position was not unexpected since this newly inserted glutamine is the immediate neighbor of the IgG1 glycosylation site (N297). Our observation is consistent with the sugar moiety preventing mTG reactivity at Y296Q due to steric hindrance, similarly to the well-established lack of reactivity of the native Q295 residue in the context of glycosylated antibody.^16^ Glycosylated WT trastuzumab, negative control S254Q (no difference in reactivity compared to WT), and weakly hFc-reactive K340Q (0.4-fold WT signal) and P445Q (0.2-fold WT signal) trastuzumab variants did not lead to observable conjugation of dansylcadaverine by mTG under the conditions tested (Fig. 2). The weakly hFc-reactive M252Q and V282Q trastuzumab variants also resulted in weak reactivity (1.5-fold WT signal) in the context of trastuzumab. This confirms that the general trend of reactivity determined using the hFc was transposable to the full-length, glycosylated antibody.

We observed that activation of mTG by removal of its pro-sequence using trypsin instead of Dispase® was essential for efficient labeling of amino molecules to the I253Q variant of trastuzumab.^23^ We did not observe this distinction when using trastuzumab that is C-terminally labeled with the LLQG Q-tag (not shown). We speculate that the two extra amino acids (Phe42 and Arg43) remaining at the site of cleavage following Dispase® activation^23^ may prevent efficient conjugation by mTG at the I253Q site.

### A degree of conjugation of 2 is achieved by mTG conjugation on trastuzumab I253Q

The DoC of the native and the I253Q trastuzumab with two distinct amino substrates was analyzed by hydrophobic interaction chromatography (HIC), using freshly prepared mTG and antibodies (Fig. 2). A DoC of 2 is expected upon conjugation of one amino molecule on each heavy chain of trastuzumab. The amino fluorophore substrate dansylcadaverine was used to visually assess reactivity and the amino substrate *N*-[(1*R*,8*S*,9*s*)-bicyclo[6.1.0]non-4-yn-9-ylmethyloxycarbonyl]-1,8-diamino-3,6-dioxaoctane (amino-BCN) was applied for strain-promoted alkyne–azide cycloaddition (SPAAC). A DoC of 1.7 was determined for conjugation with either dansylcadaverine or amino-BCN, demonstrating that these amino substrates similarly procure high reactivity with mTG, approaching the maximal expected DoC of 2 (Fig. 2 and S2†). The positive control, LLQG-tagged trastuzumab, resulted in a DoC of 0.9 with dansylcadaverine and 0.7 with amino-BCN (Fig. 2 and S2†). This tag has been used in different contexts showing reliable reactivity, although extent of reactivity was shown to vary depending on the location of the tag.^18,24,25^ Under these conditions, it did not reach the extent of conjugation observed with the I253Q trastuzumab, potentially reflecting the greater reactivity of the glutamine in the context of the I253Q variant of trastuzumab than in the context of the LLQG-tag on trastuzumab. The negative control, glycosylated WT trastuzumab, resulted in a DoC of 0.3 for conjugation with dansylcadaverine, consistent with the weak labeling observed on SDS-PAGE; no reaction was detected with amino-BCN (Fig. 2 and S2†).

Encouraged by these results, we repeated the conjugation procedure to further quantify DoC by mass spectrometry analysis, using freshly prepared mTG and antibodies (Fig. 2). Analysis by LC-MS allowed observation of exhaustive conjugation of the I253Q trastuzumab variant with dansylcadaverine (DoC = 2) (Fig. 2).

**Fig. 3:**
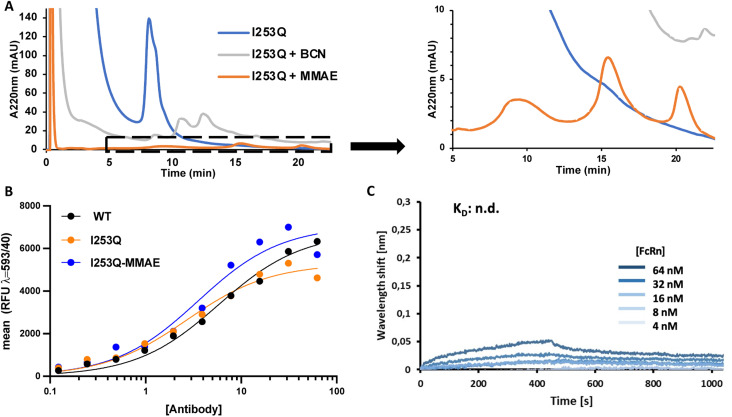
Binding affinities of trastuzumab variant I253Q to HER2 receptor and FcRn. (A) HIC chromatogram of ADC synthesis with enzymatic conjugation of amino-BCN followed by SPAAC conjugation of azido-MMAE. DoC was calculated from the area under the peak of the corresponding species. (B) Dissociation constants for the HER2 receptor were determined on SK-BR-3 cells by flow cytometry. The *K*_D_ values of 5.3 nM (WT), 3.3 nM (I253Q), and 5.0 nM (I253Q-MMAE conjugate) were determined by nonlinear regression using GraphPad prism 8.0. (C) Binding of I253Q trastuzumab variant to FcRn was measured at pH 6.0 using bilayer interferometry (BLI). No binding was observed.

MTG conjugation of the I253Q variant of trastuzumab was further investigated for the synthesis of an ADC by a two-step chemoenzymatic approach. Enzymatic conjugation of amino-BCN was followed by protein A purification to remove excess amino-BCN and mTG. Chemical conjugation of the BCN-conjugated antibody with N_3_-PEG_3_-vc-PAB-MMAE proceeded *via* SPAAC reaction. The progress of each reaction was monitored by HIC (Fig. 3). The mTG-conjugation of BCN resulted in the same conjugation efficiency (DoC = 1.7) as obtained above (Fig. 2) and MMAE conjugation was determined to be exhaustive. This demonstrates that the newly introduced glutamine is an addressable site for indirect conjugation of a cytotoxic payload.

To verify that the properties of the ADC resulting from conjugation at the newly introduced Q253 were not disrupted by substitution and subsequent conjugation with amino-BCN-MMAE, the binding affinities of the resulting ADC to HER2 and FcRn receptors were determined. Determination of ADC affinity for HER2 was analyzed by flow cytometry using the HER2-overexpressing SK-BR-3 cells, whereas binding to the FcRn receptor was measured by biolayer interferometry using non-conjugated antibody variants (Fig. 3). HER2 receptor binding affinity was not significantly perturbed either by introduction of the I253Q substitution (*K*_D_ = 3.3 nM) or by conjugation to BCN-MMAE (*K*_D_ = 5.0 nM) relative to WT trastuzumab (*K*_D_ = 5.3 nM) (Fig. 3). This was expected since all modifications were undertaken in the Fc region of the antibody, which is not the primary driver of affinity.

Association and dissociation of antibody variants to FcRn were measured at pH 6.0 and pH 7.4, respectively; those conditions are typically used to elicit the known pH dependence of FcRn binding to WT IgG in the cellular context of the lysosome (association, pH 6) and in circulation (dissociation, pH 7.4).^26,27^ FcRn binding to trastuzumab, both for WT trastuzumab and the I253Q variant. Specifically, the WT trastuzumab exhibited concentration-dependent, specific binding with an equilibrium dissociation constant of 0.8 μM (ESI Fig. S3†), consistent with previous reports^27^ whereas the I253Q variant of trastuzumab was identical to the negative control, where no specific binding is observed (Fig. 3C and S3†). Indeed, it has been shown that interaction with FcRn is abolished by the I253Q substitution in IgGs.^27^ This implies that the mechanism of antibody recycling upon nonspecific intake cannot be achieved, eventually leading to a shorter *in vivo* half-life. Interestingly, FcRn-mediated antibody recycling can be maintained in a bispecific antibody by introducing FcRn-binding enhancing mutations in one Fc monomer, where the second Fc monomer is non-functional with respect to FcRn binding.^28^ This suggests a path forward to FcRn-mediated antibody recycling in conjunction with the I253Q substitution. Cytotoxicity and specificity of MMAE-conjugated I253Q trastuzumab are maintained.

**Fig. 4:**
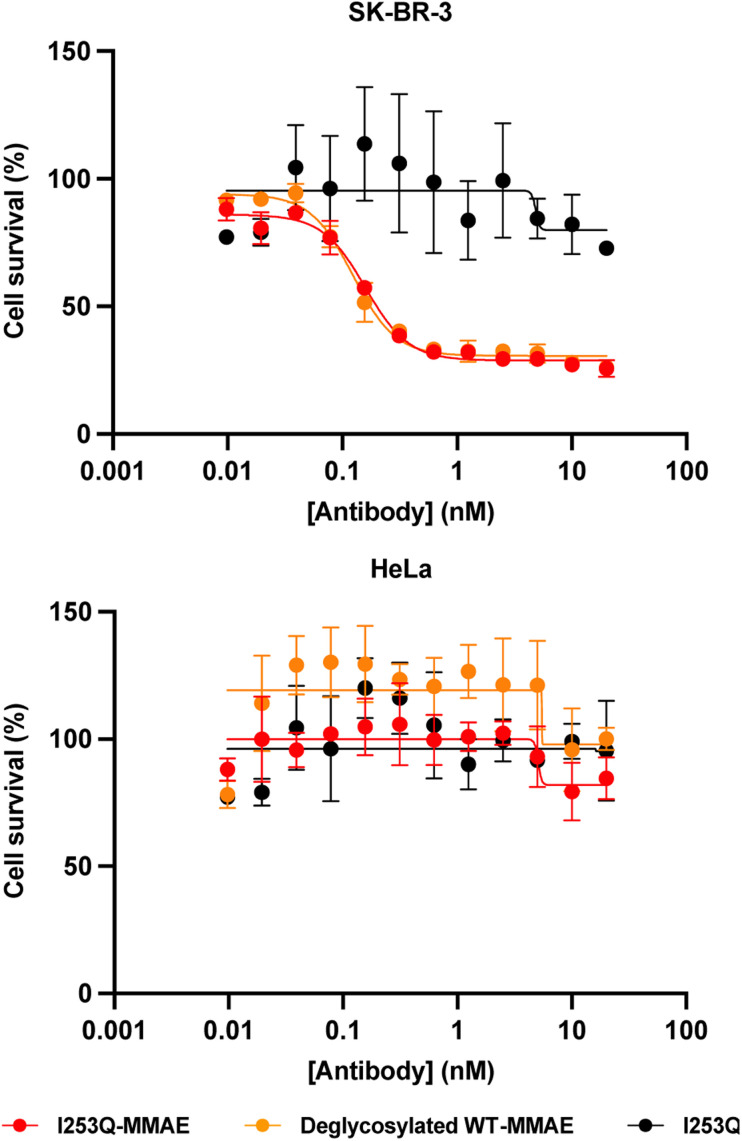
Cytotoxicity of MMAE-conjugated trastuzumab I253Q variant. Serial dilutions of trastuzumab variants (0.01 nM to 20 nM) were incubated for 3 days on SK-BR-3 (HER2^+^) cells and HeLa (HER2^−^) cells, in triplicate. Cell proliferation was assessed with CellTiter96® AQ_ueous_ (Promega). The proliferation of untreated cells was set to 100% survival. Normalized absorbance values were plotted against trastuzumab variant concentrations, and the IC_50_ values were determined using GraphPad Prism 8. Results are shown as mean ± SEM. In SK-BR-3 cells, IC_50_ (I253Q-MMAE) = 0.16 ± 0.01 nM; IC_50_ (deglycosylated WT-MMAE) = 0.12 ± 0.01 nM.

The potency and specificity of the MMAE-conjugated I253Q trastuzumab variant were evaluated by cytotoxicity assays. HER2^+^ SK-BR-3 cells and HER2^−^ HeLa cells were treated with serial dilutions of the conjugated antibody for 72 h to evaluate the cytotoxicity and the specificity of the ADC, respectively. Deglycosylated WT trastuzumab conjugated to MMAE using mTG by the same method served as a positive control (DoC = 1.8) and the unconjugated I253Q trastuzumab as a negative control. Cell proliferation assays on target SK-BR-3 cells (HER2^+^) showed no significant difference in cytotoxicity of the MMAE-conjugated I253Q variant (IC_50_ = 160 pM) and the positive control MMAE-conjugated deglycosylated WT trastuzumab (IC_50_ = 120 pM) (Fig. 4). Neither of the MMAE-conjugated antibodies was cytotoxic toward HeLa cells (HER2^−^), demonstrating that the HER2 receptor is necessary for efficient uptake of the ADC (Fig. 4). The unconjugated I253Q antibody had no cytotoxic effect on either cell type. These results indicate that the MMAE-conjugated I253Q variant of trastuzumab retained the same potency and specificity as MMAE-conjugated deglycosylated WT trastuzumab.

## Conclusions

We have demonstrated that incorporation of the I253Q substitution into the heavy chain of an IgG1 antibody produced an addressable site for mTG-mediated conjugation. The bioconjugation was compatible with antibody glycosylation. As the I253 residue is conserved in all human IgG subtypes,^29^ this conjugation approach should be directly applicable to all other human IgGs. Direct conjugation of an amino fluorophore and chemoenzymatic ADC synthesis were both successful, resulting in a maximal DoC of 2. The *in vitro* cytotoxicity of MMAE-conjugated I253Q trastuzumab is similar to that obtained with mTG-conjugated deglycosylated WT trastuzumab, yet conservation of glycosylation on the antibody promises to open avenues for applications where solubility and stability are critical.

This conjugation method is appropriate for any application that does not require binding to FcRn, such as cellular imaging, fluorescence microscopy, western blotting or ELISA.^30,31^ In its current form, disrupted binding to FcRn limits therapeutic applications of the I253Q conjugates since FcRn-mediated half-life extension is crucial for antibody pharmacokinetics. As mentioned above, bispecific antibody design could be envisaged to include the I253Q mutation in one of the two Fc monomers to benefit from its chemoenzymatic modification without largely compromising half-life.^32^ Although beyond the scope of this work, the half-life of the I253Q-based ADC and the potential to include it in a bispecific antibody design in combination with enhanced FcRn binding should be investigated in subsequent *in vivo* studies.

In conclusion, our straightforward approach allows mTG-mediated site-specific conjugation of fully glycosylated antibodies with minimal sequence alteration. As the binding affinity for the HER2 receptor is maintained, the resulting conjugate is appropriate for many imaging and binding applications.

## Abbreviations

ADC: Antibody–drug conjugate
DoC: Degree of conjugation
ESI-MS: Electrospray ionization mass spectrometry
HER2: Human epidermal growth factor receptor-2
hFc: Human crystallizable fragment
HIC: Hydrophobic interaction chromatography
FcRn: Neonatal Fc receptor
LLQG: Trastuzumab C-terminally tagged with the Leu-Leu-Gln-Gly peptide
MMAE: Monomethyl auristatin E
mTG: Microbial transglutaminase
SPAAC: Strain-promoted alkyne–azide cycloaddition
WT: Wild-type

## Conflicts of interest

There are no conflicts to declare.

## Supplementary Material

RA-012-D2RA05630E-s001
