# Supplementary material for: Tag-free, specific conjugation of glycosylated IgG1 antibodies using microbial transglutaminase
Source: RSC Adv. 2022 Nov 22;12(52):33510–5. doi: 10.1039/d2ra05630e (PMC9680618; doi:10.1039/d2ra05630e)
Supplement: RA-012-D2RA05630E-s001 [file RA-012-D2RA05630E-s001.pdf]

Electronic Supplementary Material (ESI) for RSC Advances.
This journal is © The Royal Society of Chemistry 2022

Supporting information

Tag-Free, Specific Conjugation of Glycosylated IgG1 Antibodies Using Microbial Transglutaminase

Adem Hadjabdelhafid-Parisien, Sebastian Bitsch, Arturo Macarrón Palacios, Lukas Deweid, Harald Kolmar & Joelle N. Pelletier.

## Methods

### Materials

Unless otherwise stated, the materials were purchased from Carl Roth GmbH.

### Site-directed mutagenesis

Template DNA (pTT5 trastuzumab heavy chain and pTT5 trastuzumab light chain) was kindly provided by Merck KGaA (Darmstadt, Germany). Glutamine-coding sequences were introduced at specific positions in the pTT5 trastuzumab heavy chain plasmid by site-directed mutagenesis. The primers used are provided in **Supporting Table 1**. Template (98 ng), dNTPs (200 µM each), primers (0.5 µM each), HF green buffer and Phusion® polymerase (Thermo Fischer) and nuclease-free $H_2O$ were made up to a final volume of 50 µL. PCR was initiated with denaturation at 98 °C for 30 s, followed by amplification with 30 cycles of denaturation at 98 °C for 30 s, annealing at 65 °C for 30 s, and polymerization at 72 °C for 3 min. PCR was terminated with extension at 72 °C for 5 min. The template was digested with 2 U of Dpn1 restriction enzyme (Thermo Fischer) at 37 °C for 16 hours, then deactivated at 80 °C for 20 minutes. PCR products were purified with PCR cleanup kits (Promega) according to the manufacturer's instructions. Purified PCR products were transformed into DH5α electrocompetent cells and selected on double yeast-tryptone media (dYT)-agar + Amp (100 µg/mL) and incubated for 16 h at 37 °C. Single colonies were used to inoculate 5 mL of dYT + Amp for 16 h at 37 °C with shaking and plasmids were isolated using a mini-prep kit (Promega) according to the manufacturer's instructions. Isolated plasmids were analyzed by DNA Sanger sequencing at Microsynth Seqlab GmbH (Germany) or at *plateforme de séquençage Génome Québec*. Sequence-confirmed colonies were propagated in 50 mL dYT + Amp medium. Plasmids were isolated using a midi-prep kit (Promega) according to the manufacturer's instructions.

### Antibody expression and purification

Suspension HEK Expi293F$^{TM}$ cells (Gibco) were used for antibody production. Cell count and viability were assessed using a hemocytometer. Plasmids encoding the wild-type (WT) or modified heavy chain and WT light chain (25 µg each) were added to 2.5 mL NaCl (150

mM) and 200 µL polyethyleneimine (PEI) solution. DNA-PEI complexes were mixed by vortexing and incubated for at least 15 min at room temperature. For transient transfection, freshly cultured cells were adjusted to a cell titer of 2.5 × $10^6$ cells for a final volume of 30 mL. The DNA-PEI solution was added dropwise to the cells while gently shaking. The cells were supplemented with 825 µL of 20% (w/v) tryptone 16-18 h post-transfection. Antibody expression was performed for 5 days at 37 °C, 110 rpm, and 8% $CO_2$.

Production was stopped by centrifugation at 1,000 × *g* for 10 min and the supernatant was sterilized by filtration before purification. The cell culture supernatant was diluted with an equivalent volume of PBS and applied at a flow rate of 1 mL/min to a 1-mL Hi-Trap Protein A column (GE Healthcare) pre-equilibrated with at least 5 CV of PBS (pH 7.4). The column was subsequently washed with 5-10 column volumes of PBS buffer and the protein was recovered by isocratic elution (0.1 M sodium citrate pH 3) in 1-mL fractions were recovered into tubes containing 200 µL of 1 M Tris-HCl (pH 9). Protein fractions were pooled according to the $A_{280}$ peak and dialyzed against 5 L of PBS buffer (pH 7.4) overnight at 4°C with stirring. Protein was concentrated to >2 mg/mL using an Amicon 50 kDa MWCO (Merck Millipore). Protein concentration was determined at $A_{280}$ using a molar extinction coefficient of 2.1 x$10^5$ $M^{-1}$ $cm^{-1}$ and an approximate molecular weight of 150 kDa. Protein fractions were resolved by migration on 15% SDS-PAGE analysis to assess protein purity.

**Production and purification of mTG**

Microbial transglutaminase was produced and purified as described previously [1]. The plasmid pET20b-FRAP-mTG was kindly provided by Professor M. Pietzsch (Martin-Luther-Universität, Halle-Wittenberg, Germany) [2]. The specific activity was determined by the hydroxamate test and purity was assessed by SDS-PAGE analysis[3]. Specific activity was between 5-30 U/mg and purity was over 80% (Fig. S4).

**mTG conjugation assays**

For conjugation assays, trastuzumab glutamine variants (1 mg/mL) were incubated with 5 mM of the amino substrate: dansylcadaverine or N-[(1R,8S,9s)-bicyclo[6.1.0]non-4-yn-9-ylmethyloxycarbonyl]-1,8-diamino-3,6-dioxaoctane (amino-BCN) (Sigma Aldrich), and mTG (6 U/mg of antibody) at 37 °C, with shaking at 600 rpm. After 24 h incubation, reactions were stopped by the addition of mTG blocker (Zedira) and analyzed by SDS-PAGE and hydrophobic interaction chromatography. Conjugation products were confirmed by LC-MS.

**Click-chemistry reactions**

BCN-conjugated trastuzumab was purified over protein A spin column (GE Healthcare) according to the manufacturer's instructions. Azide-$PEG_3$-vc-PAB-MMAE (MedChemExpress, 5 eq. per site) was added to the purified BCN-conjugated trastuzumab

(1 mg/mL) and incubated for 12 h at room temperature. Excess unconjugated MMAE was removed using an Amicon Ultra-0.5 (MWCO 10 kDa, Millipore Sigma), washing three times with PBS.

**Visualization of reactivity by SDS-PAGE analysis**

SDS-PAGE analysis was performed for rapid visualization of reactivity with dansylcadaverine. An aliquot (5 µg) of reactions and their corresponding negative control in reducing SDS gel loading dye were denatured at 98 °C for 10 min and resolved by migration on 10% SDS-PAGE. Gels were run at 200 V until elution of bromophenol blue. Before Coomassie staining, fluorescence was revealed using a GelDoc-It imaging system (UVP Imaging) with an emission filter at 590 nm.

**Hydrophobic interaction chromatography (HIC)**

HIC was performed to assess the degree of conjugation (DoC) of the antibody variants with dansylcadaverine and BCN. A 2.5 mm × 4.6 mm × 35 mm TSKgel Butyl-NPR column (Tosoh Bioscience), fitted on an HPLC 1260 Infinity device (Agilent) equipped with a DAD detector was used for the analysis. Samples (35 µg) were injected onto the column and separation was performed using a flow rate of 0.9 mL/min using a linear gradient of 0% to 100% eluent B over 35 min (eluent A: 1.5 M ammonium sulfate, Tris-HCl pH 7.5, eluent B: Tris-HCl pH 7.5) to determine reactivity of all variants with dansylcadaverine. BCN and MMAE conjugation to I253Q and WT trastuzumab were analyzed using a gradient of 0 to 100% over 20 min. Detection was performed at 220 nm and the area under the peak was used to determine the degree of conjugation.

**HER2 receptor cell binding assay**

Flow cytometry was used to determine the binding affinity of glutamine-reactive variants of trastuzumab on HER2-overexpressing SK-BR-3 cells. Dilutions, incubation and washing steps were performed at 4 °C with PBS (pH 7.4) containing 1% BSA. Serial dilutions of the antibody (250 nM to 0.12 nM) were incubated for 1 h with $2 \times 10^5$ cells. Phycoerythrin-labeled goat anti-human IgG Fc antibody (1:75 dilution) (Invitrogen) was added and incubated for 30 min. A BDInflux cell sorter and BD FACS software were used to determine cell fluorescence over $5 \times 10^4$ events.

**In vitro cytotoxicity**

SK-BR-3 and HeLa cells were used to determine the cytotoxicity and specificity of the I253Q trastuzumab glutamine variant conjugated to MMAE. In a 96-well plate, cells were seeded at 5 000 viable cells/well the day before the experiment. Serial dilutions (20 nM to 0.02 nM) of antibodies were incubated with either of the cell types for 72 h at 37 °C, 5% $CO_2$. The CellTiter96® $AQ_{ueous}$ cell proliferation assay (Promega) was used to assess cell viability according to the manufacturer's instructions.

**FcRn binding**

Affinity determination was performed on the Octet® RED96 system (ForteBio, Molecular Devices) using Octet® anti-human Fab-CH1 2nd generation (FAB2G) biosensors. Sensors were soaked in PBS pH 7.4 for 10 min, and subsequently loaded with 10 µg/ml of the antibody of interest in PBS pH 7.4 until a response of 0.7 to 1 nm was reached, followed by 2 min of quenching in kinetics buffer pH 6.0 (FortéBio). Association of decreasing concentrations of FcRn (kindly provided by Merck KGaA (Darmstadt, Germany)), ranging from 64 nM – 4 nM in a two-fold dilution series, was measured in PBS pH 6.0 for 7 min. As a negative control, kinetics buffer pH 6.0 was used instead of FcRn solution. Due to the pH-dependent binding mechanism of FcRn, dissociation was measured in PBS pH 7.4 for 10 min. All measurements were performed at 30 °C and 1,000 rpm. Binding kinetics were determined based on Savitzky-Golay filtering and a 1:1 Langmuir binding model using the respective negative control.

**LC-MS**

Freshly prepared antibodies and mTG were used for MS ana

lysis. LC-MS separations of intact proteins were performed on a TOF 6224A instrument coupled to an HPLC 1260 Infinity, both from Agilent Technologies. The chromatographic column was an Aeris widepore XB-C8, 3.6 µm, 4.6 x 100 mm column from Phenomenex. Eluents consisted of 0.1% formic acid in water (eluent A) and acetonitrile (eluent B). Elution was performed at 0.4 mL/min with a gradient from 10% B to 70% B over 9 min and a total run time of 15 min. The electrospray ionization source was used in positive ion mode and mass spectra were acquired from m/z 100 to 3200. Agilent Mass Hunter software was used for instrument control, data acquisition and analysis (Bioconfirm module).

**Supporting Table 1. Primers for mutagenesis on pTT5 trastuzumab heavy chain.**

| Position | Forward[a] | Reverse |
|---|---|---|
| M252Q | CACCACCACG**CAG**GTCACTTCGGGG | AGGATACCCTGCAGATCAGCCGGAC |
| I253Q | ATACCCTGATG**CAG**AGCCGGACCCC | ATCCACCACCACGCAGGTCACTTCG |
| S254Q | CCCTGATGATC**CAG**CGGACCCCCGA | CACATCCACCACCACGCAGGTCACT |
| V282Q | ACGTGGACGGC**CAG**GAAGTGCACAA | TTCCTCTCTGGGCTTGGTCTTGGCG |
| Y296Q | GAGAGGAACAG**CAG**AACAGCACCTA | CACTGTCAGCACGGACACCACCCGG |
| K340Q | TCAGCAAGGCC**CAG**GGCCAGCCCCG | AGGCAGTGTGTACACCTGGGGTTCG |
| P445Q | TGAGCCTGAGC**CAG**GGCTGAGGATC | TATTAGCCAGAGGTCGAGGTCGGGG |

[a] The mutagenic codon is indicated in bold.

**Supporting Table 2. Trastuzumab variant purification yields.**

| Variant | Yield (mg) |
|---|---|
| WT | 1.7 |
| LLQG | 1.5 |
| M252Q | 0.9 |
| I253Q | 1.5 |
| S254Q | 1.2 |
| V282Q | 1.0 |
| Y296Q | 1.2 |
| K340Q | 1.3 |
| P445Q | 0.8 |

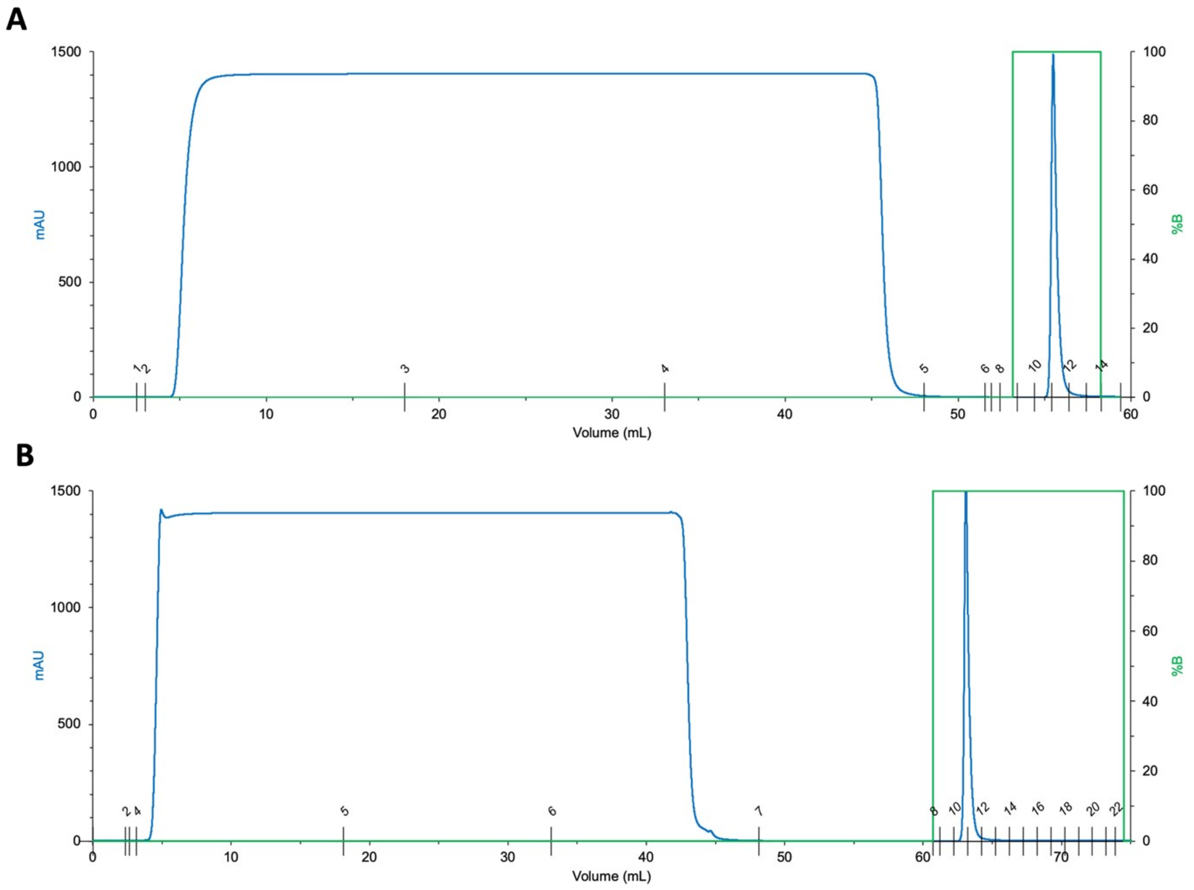


**Supporting Figure 1. Protein A purification chromatograms of A) WT and B) I253Q trastuzumab variants.** Blue curve represents absorbance at 280 nm and green curve shows percentage of B pump buffer. Fractions are indicated by ticks on x-axis.

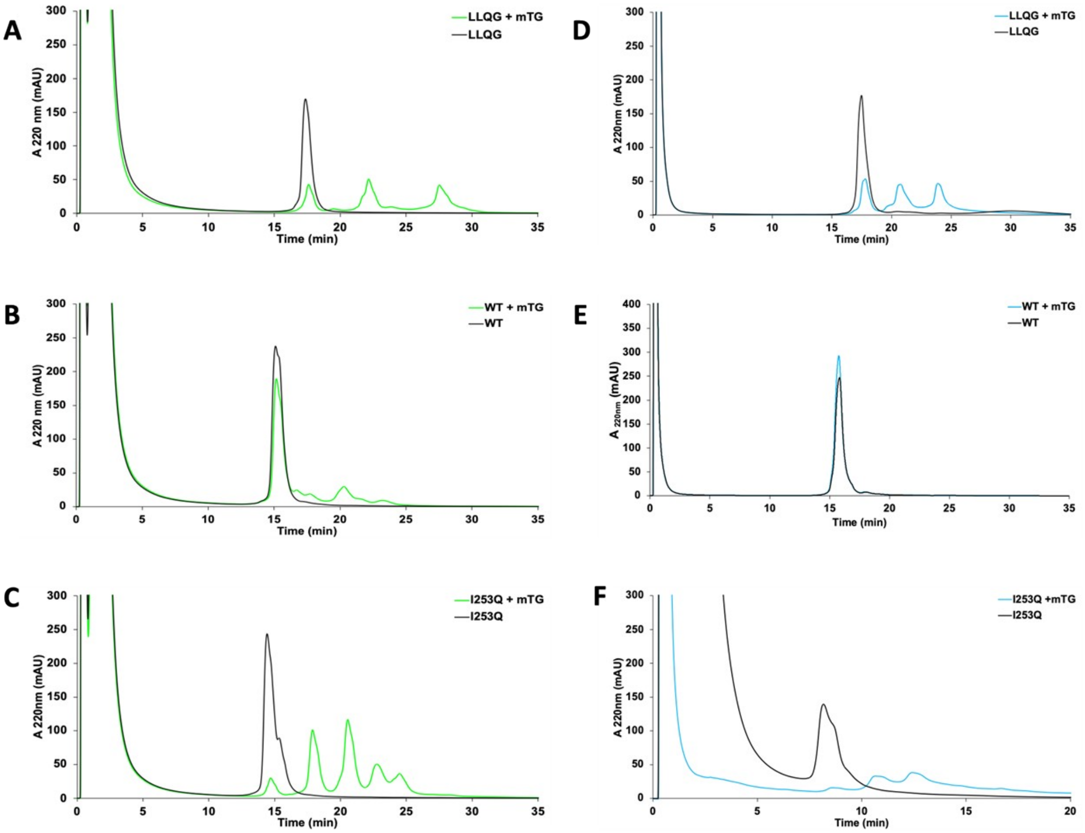


**Supporting Figure 2. HIC chromatograms for conjugation of trastuzumab with amino-substrates dansylcadaverine (A-C; green traces) or amino-BCN (D-F; blue traces).** Conjugation of LLQG-tagged (A, D), WT (B, E) or I253Q trastuzumab (C-F) are shown.

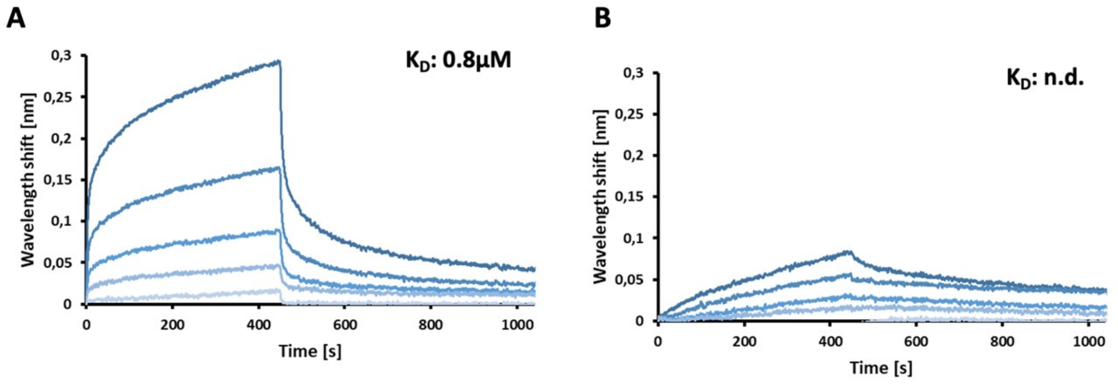


**Supporting Figure 3. FcRn binding evaluation by BLI.** A. WT trastuzumab FcRn binding was evaluated at $K_D$ = 0.8 µM. B. PBS was used to evaluate non-specific binding to FcRn.

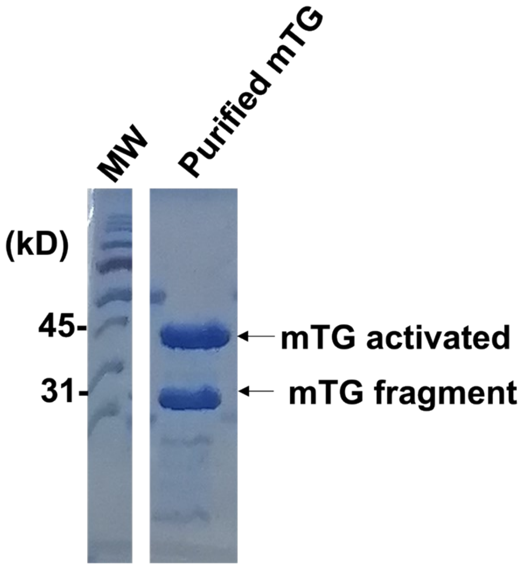


**Supporting Figure 4. Purity evaluation of purified mTG.** MW, molecular weight marker, Broad-range BioRad.

**Supporting references**


[1] Deweid, L., Hadjabdelhafid-Parisien, A., Lafontaine, K., Rochet, L. N. C., Kolmar, H., and Pelletier, J. N. (2020) Glutamine-walking: Creating reactive substrates for transglutaminase-mediated protein labeling, In *Methods Enzymol.*, Academic Press.

[2] Marx, C. K., Hertel, T. C., and Pietzsch, M. (2007) Soluble expression of a pro-transglutaminase from *Streptomyces mobaraensis* in *Escherichia coli*, *Enzyme Microb Tech 40*, 1543-1550.

[3] Folk, J. E., and Cole, P. W. (1966) Mechanism of action of guinea pig liver transglutaminase. I. Purification and properties of the enzyme: identification of a functional cysteine essential for activity, *J Biol Chem 241*, 5518-5525.
